# A deep learning model based on ultrasound imaging to differentiate malignant from benign pleural effusion: a multicenter cohort study

**DOI:** 10.1186/s12931-026-03574-w

**Published:** 2026-02-11

**Authors:** Chang-Wei Wu, Chia-Suan Yu, Yen-Lin Chen, Po-Chih Kuo, Meng-Rui Lee, Jann-Yuan Wang, Chao-Chi Ho, Jin-Yuan Shih, Hao-Chien Wang

**Affiliations:** 1 Division of Pulmonary and Critical Care Medicine, Department of Internal Medicine, National Taiwan University Hospital, Hsin-Chu branch, Taipei, Hsin-Chu, Taiwan; 2 Department of Computer Science, National Tsing Hua University, No. 101, Kuang Fu Rd, Sec.2, , Hsinchu, Hsinchu 300 Taiwan; 3 Division of Pulmonary and Critical Care Medicine, Department of Internal Medicine, National Taiwan University Hospital, #7, Zhongshan South Rd., Zhongzheng Dist., Taipei, 100226 Taiwan; 4 Department of Medicine, National Taiwan University Cancer Center, Taipei, Taiwan

**Keywords:** Malignant pleural effusion, Thoracic ultrasound, Deep-learning, Diagnosis

## Abstract

**Background:**

Thoracentesis is required for malignant pleural effusion (MPE) diagnosis. However, it is an invasive procedure and carries risks. It remains unknown whether deep learning using ultrasound images could become a non-invasive diagnostic approach.

**Methods:**

Patients with pleural effusion detected by thoracic ultrasound and received diagnostic thoracentesis were retrospectively collected from two sites. The internal cohort collected patients from the National Taiwan University Hospital (NTUH) Hsin-Chu branch (2014–2021), whereas the external cohort collected patients from NTUH (2020–2021). The MPE was confirmed by cytopathology reports, while benign pleural effusion was ascertained by negative cytology and compatible clinical courses. A convolutional deep learning model was used to identify MPE. Performance metrics included accuracy, F1 score, sensitivity, specificity and the area under the receiver operating characteristic curve (AUC).

**Results:**

A total of 601 and 144 patients from the internal cohort and the external cohort were used for model development. The model achieved promising results in internal testing (accuracy = 0.750 [95% CI: 0.689–0.811], sensitivity = 0.710 [95% CI: 0.619–0.798], specificity = 0.803 [95% CI: 0.704–0.893], F1 = 0.763 [95% CI: 0.691–0.826], AUC = 0.814 [95% CI: 0.746─0.873]). After fine-tuning with small number of external images, the model achieved the following performance on the external testing set: accuracy = 0.774 [95% CI: 0.679–0.857], sensitivity = 0.818 [95% CI: 0.723–0.905)], specificity = 0.611 [95% CI: 0.389–0.846], F1 = 0.850 [95% CI: 0.776–0.913], AUC = 0.753 [95% CI: 0.596–0.885].

**Conclusions:**

Our deep learning model holds promise as a non-invasive point-of-care modality for assistance in pleural effusion diagnosis.

## Introduction

The presence of pleural effusion is common when patients encounter unresolved disease involving lung, heart, pleura, etc [1, 2]. The burden of pleural effusion on patients is significant. For instance, an estimated 1.5 million of patients suffer from pleural effusion annually in the United States [3]. Another study in China found a significant hospitalization cost of pleural effusion management, approaching 40% of mean household income for every hospitalization [4]. The disease burden of MPE is also significant, affecting approximately 1 million people globally per year, causing disabling respiratory symptoms and was associated with shortened life expectancy [5].

Identifying malignant pleural effusion (MPE) from other etiologies is an important but challenging task [3, 6, 7]. The presence of MPE indicates metastatic disease and a timely diagnosis can prompt early intervention [8, 9]. A cytopathology review on pleural effusion or pleural biopsy specimen is the gold standard for diagnosis, but the diagnosis yield is far from perfect [1, 2, 9]. Arnold et al. reported the diagnostic difficulty in diverse cancer types, with sensitivity ranging from 0 to 94.7% [10]. Porcel et al. reported a negative pleural effusion cytology result can be overturned from the second (55/214, 26%) and third thoracentesis (12/52, 23%) pleural effusion cytology result [11]. In these cases, repeated thoracentesis and testing are usually required, which leads to delayed diagnosis, unresolved symptoms, and increased risk of procedure-related complications to patients [12]. A non-invasive, rapid, and sensitive test to confirm the cause of pleural effusion is crucial.

Machine and deep learning methods have demonstrated great potential in diagnosing MPE based on models developed through chest radiography (CXR) and computed tomography (CT) [13–15]. However, no study has classified pleural effusion based on thoracic ultrasound (TUS) images developed by deep learning algorithms. The popularity and portability with ultrasound device provide an easy access to evaluate pleural effusion at resource-limited areas or at referral medical centers [16, 17]. Ultrasound also has the advantage to provide real-time images in non-invasive, cost-effective, and radiation-free approach. Previous studies relied on specialists’ interpretation of ultrasound images and clinical data to classify MPE and non-MPE [18, 19]. However, a recent meta-analysis suggested ultrasound interpretation to be a challenging task even for specialists [20]. The study showed a wide range of sensitivity and specificity regarding ultrasound echogenicity or pleura thickening for MPE diagnosis, while pleura nodularity, a specific sign of MPE, showed a poor sensitivity of 42.5%.

To further explore the diagnostic applications of ultrasound with deep learning in differentiating between malignant and benign pleural effusion, we aim to develop the first TUS image-based deep learning model to distinguish between MPE and benign pleural effusion (BPE).

## Methods

### Patient population and data collection

**Fig. 1:**
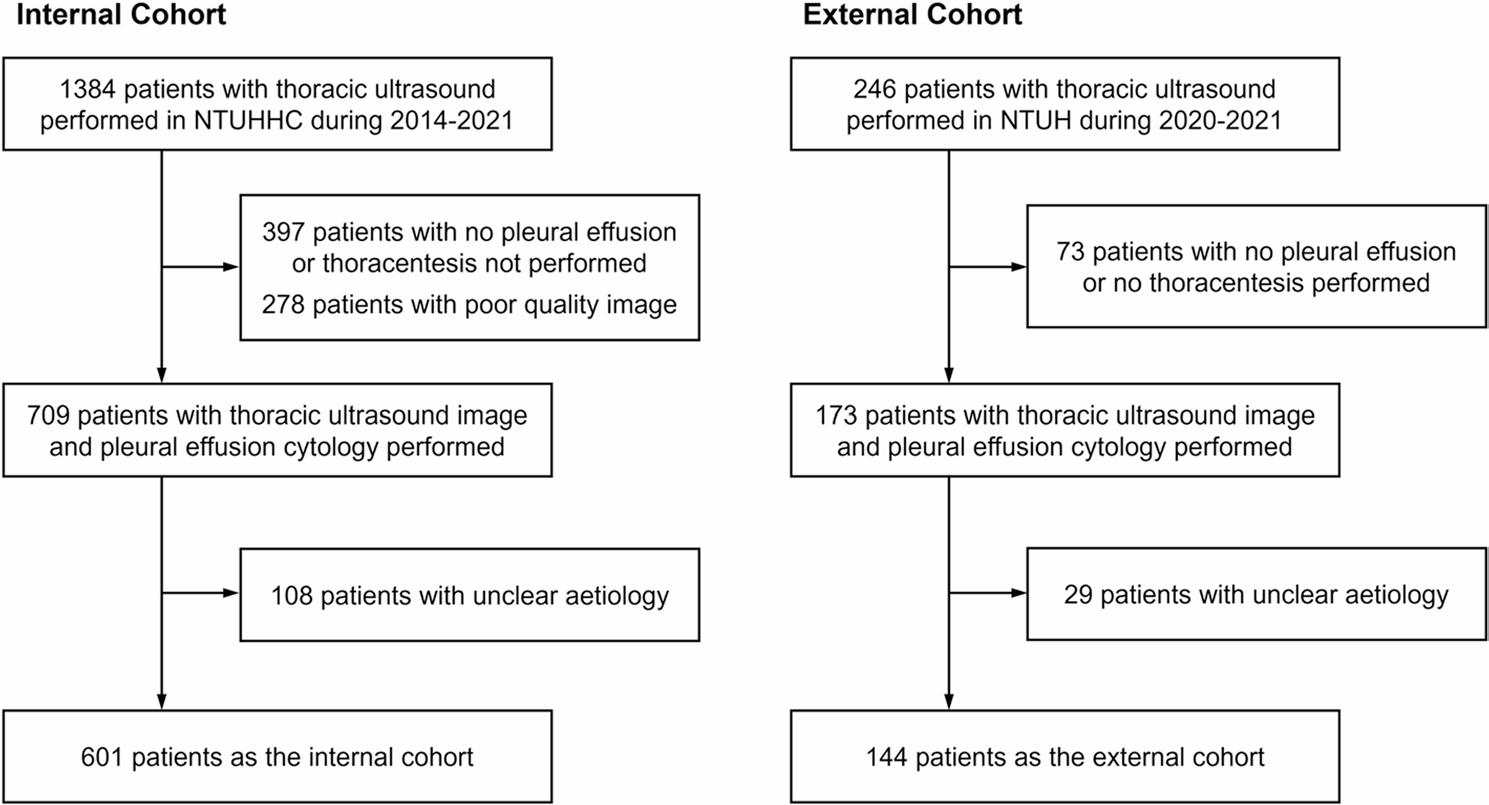
Flowchart of inclusion and exclusion process. NTUHHC, National Taiwan University Hospital Hsinchu Branch; NTUH, National Taiwan University Hospital

This study was approved by the Institutional Review Board of National Taiwan University Hospital (110-129-E and 202110052RINC) with the requirement for written informed consent waived because this study was retrospective and added no risk to participants. This retrospective cohort study was conducted at two sites: the National Taiwan University Hospital Hsin-Chu branch (internal cohort) and the National Taiwan University Hospital (external cohort) in Taiwan. The internal cohort included patients who received TUS with any pleural effusion detected between January 1, 2014, and December 31, 2021, while the external cohort included patients from November 25, 2020, to August 30, 2021. The exclusion criteria were (1) pleural effusion with unclear aetiology after the pulmonologist review and (2) unattainable pleural effusion cytology/pleura pathology or clinical information. Both cohorts followed the same exclusion criteria as shown in Fig. 1.

We collected basic demographic characteristics, underlying disease, and pleural effusion parameters from electrical medical records. The aetiology of the effusion was stringently reviewed by two pulmonologists. The definition for MPE was based on a positive pleural effusion cytology/pleura pathology. Cases classified as BPE were confirmed with a definite aetiology other than MPE (e.g., heart failure, tuberculous pleural effusion) with no malignant underlying disease, avoiding a possible false negative result due to the limitation of cytology examination. TUS images were extracted from different ultrasound machine models across sites and different timings. ALOKA ARIETTA 850 (HITACHI), ALOKA ARIETTA 750 (HITACHI), and ALOKA ARIETTA 60 (HITACHI) were used in National Taiwan University Hospital Hsin-Chu branch, while ALOKA ARIETTA 850 (HITACHI), Aplio A500 (TOSHIBA) were used in National Taiwan University Hospital. Duplicate cases were identified and only data from their first TUS record were selected. All pulmonologists in the National Taiwan University Hospital medical system follow a standard protocol of TUS. All patients were required to sit upright prior to examination unless intolerance. Convex ultrasound transducer was placed in the lowest part of posterior lung field at bilateral costophrenic angle to scan the dependent part of pleural effusion. For patients in non-sitting position, or without detectable pleural effusion within posterior view, lateral and anterior pleural cavity at costophrenic angle would be scanned. All TUS images were recorded, including ultrasound features highly suggestive of malignancy, such as pleural nodularity. All collected images were screened and only those with the presence of pleural effusion, cytology report, and accountable aetiologies were included Fig. 1. All qualified TUS images for final analysis were reviewed by pulmonologist CWW, excluding those with prominent annotation masking the region of interest and blurred anatomic structures. To ensure scientific rigor, data collection was conducted independently of the model training workflows to prevent potential biases.

### Data preprocessing

**Fig. 2:**
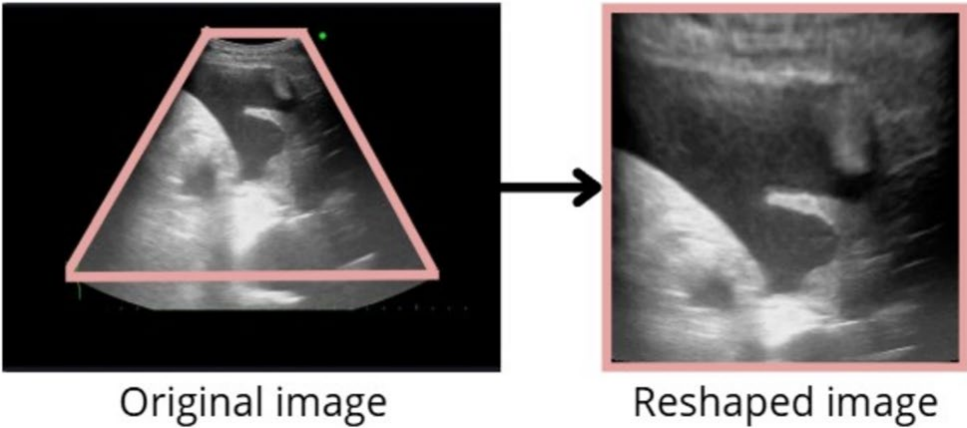
Flowchart of the standardized image processing. Original ultrasound images were first cropped to the region of interest, demarcated by a pink frame. The cropped images were then reshaped to a square aspect ratio and resized to a uniform resolution of 224 × 224 pixels

Due to the retrospective nature of this study, there was no unified ultrasound machines and device settings. We used a simple standard image processing method in our study to magnify the region of interest of the TUS image and ensure consistency. In this method, TUS images were reshaped from fan-shape to square-shape images in 224 × 224 pixels, as shown in Fig. 2. During the preprocessing phase, technicians were blinded to the labels and clinical diagnoses of the images. All images were processed using the same standardized protocol to ensure consistency.

### Model evaluation

A deep learning model (TUSnet) was initially developed using the internal cohort, consisting of 601 patients with 1706 TUS images. These TUS images were randomly stratified into internal training (*n* = 480), internal validation (*n* = 60), and internal testing (*n* = 61) datasets at an 8:1:1 ratio. TUSnet was trained on the internal training set and validated using the internal validation set, with the final evaluation performed on the internal testing set.

We applied a fine-tuning model to further enhance model performance and generalizability in the external cohort. The external cohort, consisting of 144 patients with 144 TUS images (1 TUS image per patient), were randomly stratified into an external fine-tuning cohort (*n* = 60) and external testing cohort (*n* = 84).

Performance metrics, including accuracy, area under the receiver operating characteristic curve (AUC), F1 score, sensitivity, and specificity, were reported for both models.

### Data augmentation

**Fig. 3:**
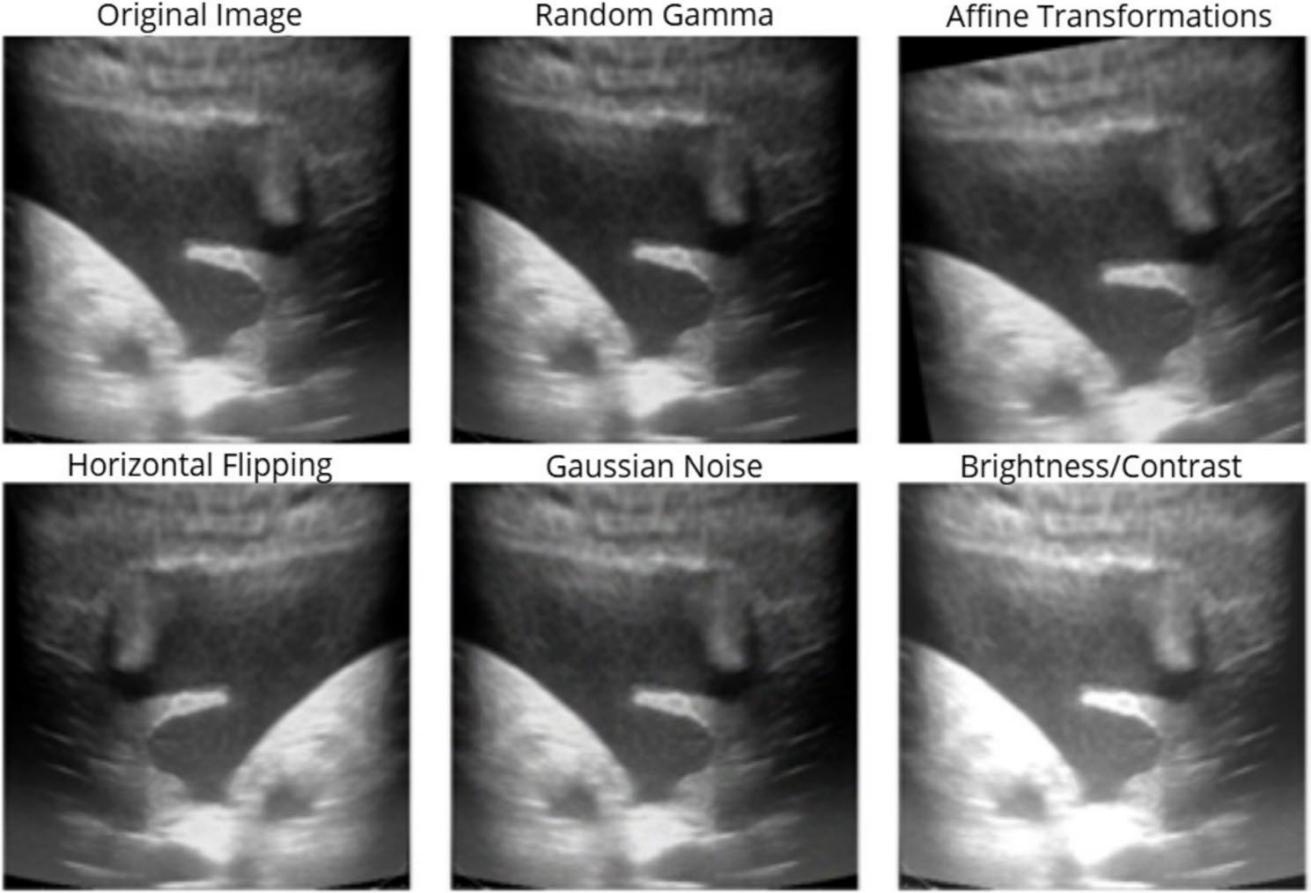
Data augmentation methods. Data augmentation was applied to the training set to prevent overfitting and increase dataset diversity. The process involved a random combination of augmentation method applied to each image, including random gamma correction, affine transforms, horizontal flipping, Gaussian noise, and brightness/contrast adjustments

We implemented a data augmentation strategy using the Albumentations library which is shown in Fig. 3 [21]. Each instance was randomly subjected (*p* = 0.5) to one of the following operations: random gamma adjustment (gamma_limit = (80, 120)), affine transformations (± 5% translation, ± 10° rotation, 0.9–1.1 scaling), horizontal flipping, Gaussian noise injection (variance limit 10.0–50.0), or brightness/contrast adjustment (limit 0.2). For the internal dataset, both benign and malignant images were augmented three times per instance. In the external fine-tuning dataset, to mitigate class imbalance, benign images were augmented four times while malignant images were augmented twice.

### Model architecture

TUSnet employs ResNet-18 as its backbone architecture, pre-trained on the ImageNet database. The model was adapted for binary classification of MPE by modifying the final fully connected layer to a two-class output layer. To preserve the pre-trained features, the first five layers were frozen during initial training on the internal cohort, while the remaining layers underwent fine-tuning. Input images were standardized to 224 × 224 pixels. The model was trained using focal loss (gamma = 2) with an initial alpha of 0.5 and optimized with the Adam optimizer at a learning rate of 5 × 10^− 5^.

**Fig. 4:**
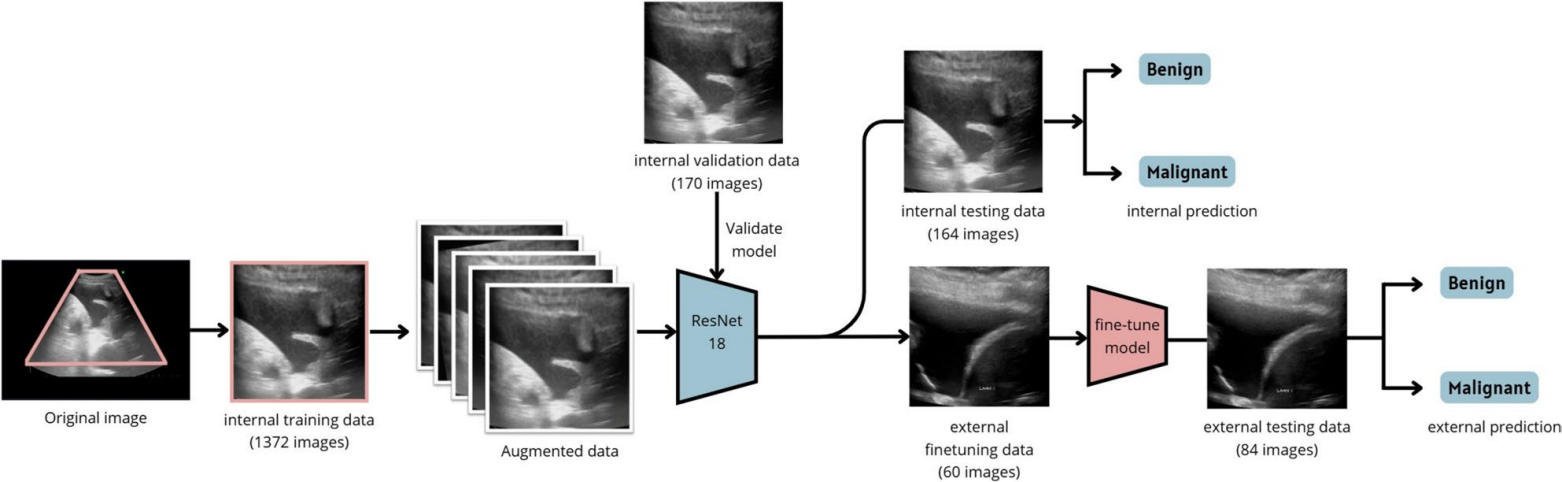
Study flowchart illustrating model development and external validation. The TUSnet model, which utilizes a ResNet-18 backbone, was developed in two primary stages. First, the model was trained on the internal cohort using standardized pre-processing and data augmentation, with its performance established through internal validation and testing. Second, to improve generalizability and adapt the model to a new data domain, the pre-trained model underwent fine-tuning on the external cohort. The final, fine-tuned model was then evaluated on the external test set for its predictive performance

For the external fine-tuning cohort, the pre-trained model was further refined using 60 external cases while all model parameters were unfrozen. A focal loss with an adjusted alpha of 0.3 was employed, and the model was trained for 1 to 10 epochs with the same optimizer and learning rate. Model performance was continuously evaluated on an independent external testing set (*n* = 84). The architectural framework is illustrated in Fig. 4.

### Statistical analysis

Basic characteristics are presented as means ± standard deviations or as numbers (percentages). Categorical variables were compared using the Chi-square test, while continuous variables were analyzed using the independent student t-test. Receiver operating characteristic (ROC) curves and AUC were generated to evaluate the model performance. A two-sided *p-*value of less than 0.05 was considered significant. Data processing was performed with Python and OpenCV and statistical analyses were performed using SPSS version 25.0 for Windows (IBM Corp., Armonk, NY, USA).

## Results

### Baseline characteristics

Baseline characteristics are demonstrated in Table 1. In the internal cohort, a total of 601 patients were enrolled (mean age: 71 years; male: 59.2%), with MPE accounting for half (49.9%, 300/601) of the cases. For external validation, an independent cohort of 144 patients was utilized (mean age: 66 years; male: 49.0%). The distribution of MPE in each cohort was 49.4%, 50%, 54.1%, 78.3% and 78.6% in the internal training, internal validation, internal testing, external fine-tuning cohort, and external testing cohort (*p* < 0.001), respectively. The proportion of unilaterality of pleural effusion, pulmonary tuberculosis was not different between the internal and external cohorts, while there were significant differences in smoking history, heart failure, pneumonia, and end-stage kidney disease.

**Table 1:** Baseline characteristics of the internal and external cohort

	Total (*n* = 745)		Internal cohort		*p* value	External cohort		*p* value	Internal and External group *p* value
		Training (*n* = 480)	Validation (*n* = 60)	Testing (*n* = 61)		Fine-tuning (*n* = 60)	Testing (*n* = 84)		
Age	70.17 ± 14.59	71.01 ± 14.95	70.45 ± 14.22	73.29 ± 13.89	0.485	66.80 ± 12.61	65.33 ± 13.39	0.508	**< 0.001**
Age > 65	495 (66.4%)	321 (66.9%)	43 (71.7%)	49 (80.3%)	0.090	33 (55.0%)	49 (58.3%)	0.690	**0.008**
Sex	411 (55.1%)	275 (57.3%)	34 (56.7%)	31 (50.8%)	0.632	24 (40.0%)	47 (56.0%)	0.059	0.135
Unilateral PLE	502 (67.3%)	310 (64.6%)	44 (73.3%)	44 (72.1%)	0.237	43 (71.7%)	61 (72.6%)	0.900	0.198
Smoker and ex-smoker	283 (37.9%)	205 (42.7%)	20 (33.3%)	23 (37.7%)	0.320	15 (25.0%)	20 (23.8%)	0.870	**< 0.001**
Heart failure	131 (17.6%)	119 (24.8%)	10 (16.7%)	15 (24.6%)	0.591	6 (10.0%)	5 (6.0%)	0.367	**< 0.001**
Pulmonary TB	10 (1.3%)	6 (1.3%)	2 (3.3%)	1 (1.6%)	0.455	0 (0.0%)	1 (1.2%)	0.396	0.696
Pneumonia	127 (17.0%)	99 (20.6%)	9 (15.0%)	10 (16.4%)	0.469	3 (5.0%)	6 (7.1%)	0.600	**< 0.001**
Liver Cirrhosis	31 (4.2%)	21 (4.4%)	4 (6.7%)	3 (4.9%)	0.727	2 (3.3%)	1 (1.2%)	0.375	0.243
ESKD	40 (5.4%)	31 (6.5%)	2 (3.3%)	5 (8.2%)	0.528	2 (3.3%)	0 (0.0%)	0.092	**0.013**
Effusion aetiology									
Malignant PLE	413 (55.4%)	237 (49.4%)	30 (50.0%)	33 (54.1%)	0.786	47 (78.3%)	66 (78.6%)	0.973	**< 0.001**
Lung cancer	354 (47.5%)	208 (43.3%)	27 (45.0%)	30 (49.2%)		34 (56.7%)	55 (65.5%)		
Other cancer	60 (8.0%)	29 (6.0%)	3 (5.0%)	3 (4.9%)		13 (21.7%)	11 (13,1%)		
Benign PLE	332 (44.5%)	243 (50.6%)	30 (50.0%)	28 (45.9%)	0.786	13 (21.7%)	18 (21.4%)	0.973	**< 0.001**
Parapneumonic	78 (10.5%)	58 (12.1%)	4 (6.7%)	7 (11.5%)	0.464	3 (5.0%)	6 (7.1%)	0.6	0.070
Empyema	31 (4.2%)	24 (5.0%)	4 (6.7%)	2 (3.3%)	0.694	0 (0.0%)	1 (1.2%)	0.396	0.018
TB pleurisy	12 (1.6%)	10 (2.1%)	2 (3.3%)	0 (0.0%)	0.406	0 (0.0%)	0 (0.0%)		0.137
Heart failure/fluid overload	159 (21.3%)	124 (25.8%)	10 (16.7%)	15 24.6%)	0.302	6 (10.0%)	4 (4.8%)	0.223	**< 0.001**
Haemothorax	8 (1.1%)	3 (0.6%)	3 (5.0%)	1 (1.6%)	0.011	0 (0.0%)	1 (1.2%)	0.396	1.000
Hepatohydrothorax	13 (1.7%)	9 (1.9%)	2 (3.3%)	2 (3.3%)	0.628	0 (0.0%)	0 (0.0%)		0.084
Atelectasis or SVC syndrome	9 (1.2%)	5 (1.0%)	2 (3.3%)	1 (1.6%)	0.337	1 (1.7%)	0 (0.0%)	0.235	1.000
Post cardiothoracic surgery	8 (1.1%)	5 (1.0%)	2 (3.3%)	0 (0.0%)	0.199	0 (0.0%)	1 (1.2%)	0.396	1.000
Inflammatory disease	10 (1.3%)	5 (1.0%)	1 (1.7%)	0 (0.0%)	0.64	0 (0.0%)	4 (4.8%)	0.086	0.108
Chylothorax	2 (0.3%)	1 (0.2%)	0 (0.0%)	0 (0.0%)	0.882	1 (1.7%)	0 (0.0%)	0.235	0.349
Asbestosis	2 (0.3%)	0 (0.0%)	0 (0.0%)	0 (0.0%)		2 (3.3%)	0 (0.0%)	0.092	0.037

Data are presented with the number and percentage of patients, n (%)

*PLE* pleural effusion, *TB* tuberculosis, *ESKD* end-stage kidney disease, *SVC* superior vena cava

### Model performance

The TUSnet model achieved the following performance metrics with standard image processing and augmentation on the internal cohort. On the internal validation set: accuracy = 0.771 [95% CI: 0.700–0.829], sensitivity = 0.839 [95% CI: 0.762–0.906], specificity = 0.688 [95% CI: 0.678–0.779], F1 = 0.800 [95% CI: 0.728–0.853], AUC = 0.811 [95% CI: 0.744–0.866]; on the internal testing cohort: accuracy = 0.750 [95% CI: 0.689–0.811], sensitivity = 0.710 [95% CI: 0.619–0.798], specificity = 0.803 [95% CI: 0.704–0.893], F1 = 0.763 [95% CI: 0.691–0.826], AUC = 0.814 [95% CI: 0.746–0.873].

After fine-tuning with 60 external images, the model achieved the following performance on the external testing cohort: accuracy = 0.774 [95% CI: 0.679–0.857], sensitivity = 0.818 [95% CI: 0.723–0.905)], specificity = 0.611 [95% CI: 0.389–0.846], F1 = 0.850 [95% CI: 0.776–0.913], AUC = 0.753 [95% CI: 0.596–0.885].

**Fig. 5:**
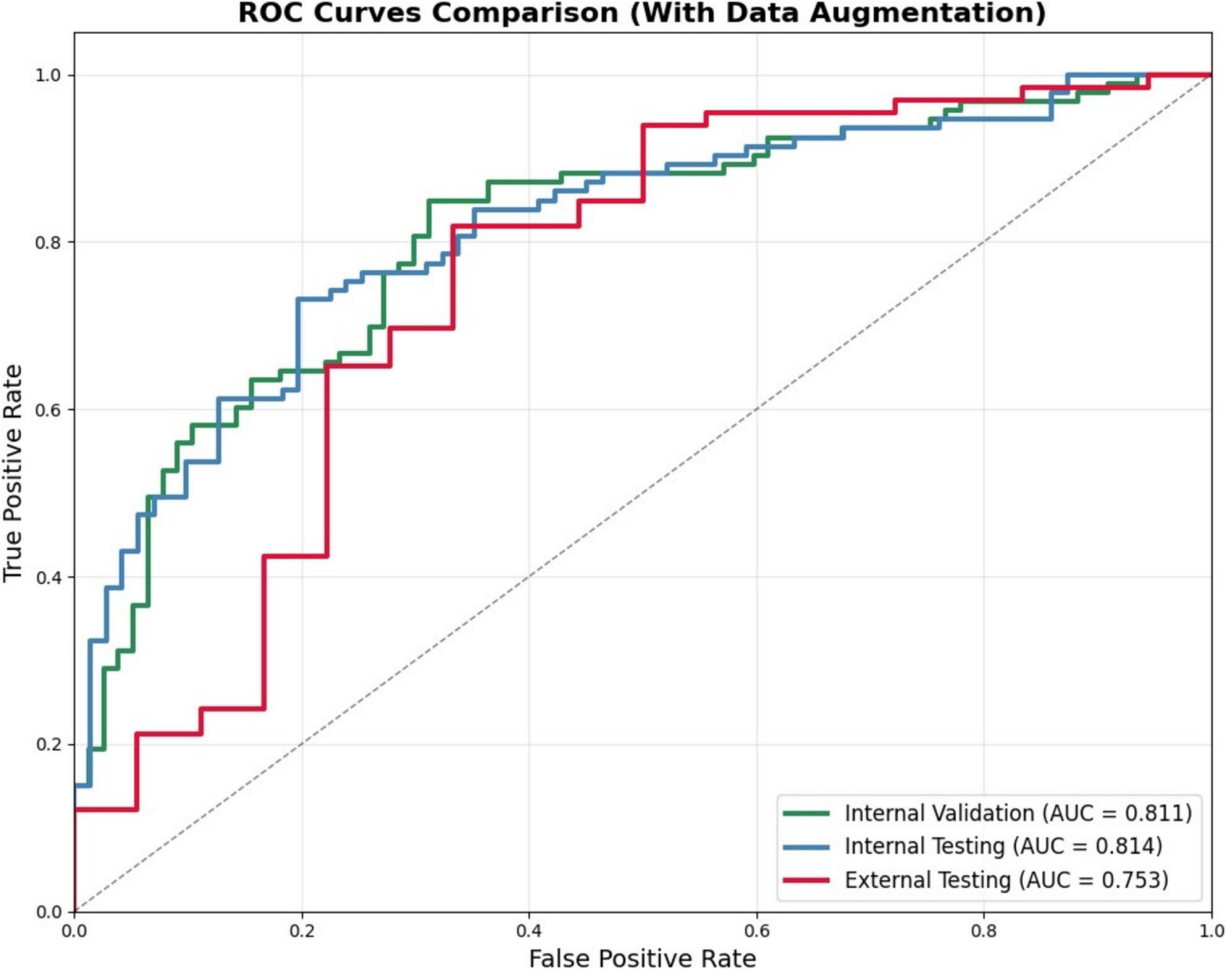
Performance of the TUSnet model. Receiver Operating Characteristic (ROC) curves illustrating the performance of TUSnet on the internal validation (green), internal testing (blue), and external testing (red) cohorts. The corresponding area under the receiver operating characteristic curve (AUC) for each cohort is provided in the legend

The complete performance metrics of TUSnet on the internal cohort and the corresponding performance metrics of TUSnet on the external fine-tuning cohort and external testing cohort are summarized in Table 2; Fig. 5.

**Table 2:** Performance metrics of TUSnet with data augmentation

Internal Validation (95% CI)	Accuracy	Sensitivity	Specificity	F1 score	AUC
	0.771 (0.700–0.829)	0.839 (0.762–0.906)	0.688 (0.678–0.779)	0.800 (0.728–0.853)	0.811 (0.744–0.866)
Internal Testing (95% CI)	0.750 (0.689–0.811)	0.710 (0.619–0.798)	0.803 (0.704–0.893)	0.763 (0.691–0.826)	0.814 (0.746–0.873)
External Testing (95% CI)	0.774 (0.679–0.857)	0.818 (0.723–0.905)	0.611 (0.389–0.846)	0.850 (0.776–0.913)	0.753 (0.596–0.885)

*AUC* area under the receiver operating characteristic curve, *CI* confidence interval

### Attention heatmap analysis

**Fig. 6:**
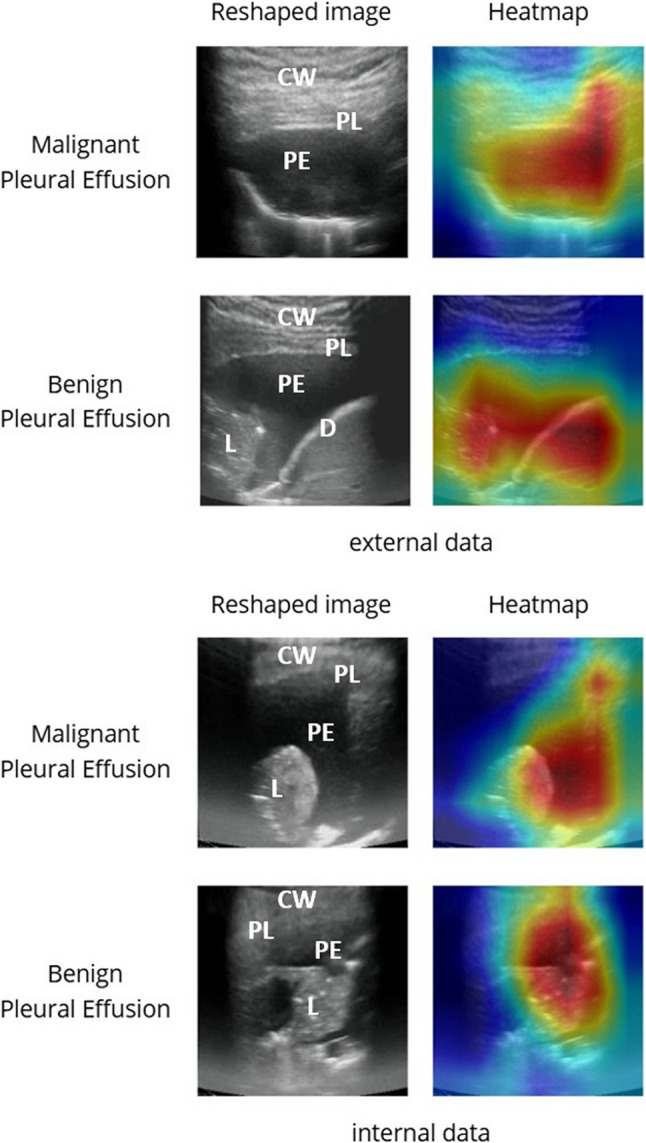
Visualization of TUSnet’s predictive rationale. Heatmap was employed to visualize the image regions most influential to TUSnet’s predictions. The figure displays attention heatmap for exemplary malignant and benign cases from both the internal and external testing sets. Each panel set includes the reshaped model input, and the corresponding heatmap, allowing for a qualitative assessment of the model’s decision-making process. Grad-CAM, Gradient-weighted Class Activation Mapping; L, Lung; D, diaphragm; PE, pleural effusion; PL, pleura; CW, chest wall

The attention heatmaps were generated to seek the importance of findings on thoracic ultrasound images. As demonstrated in Fig. 6, both visualization methods revealed high-response areas encompassing not only the pleural effusion but also the surrounding anatomic structures. A proportion of these visualization maps showed the attention more over the surrounding anatomic structures rather than the pleural effusion itself. The similar patterns observed in the attention heatmap further validate our model’s attention to these anatomically significant regions. Our deep learning model may differentiate between MPE and BPE by analyzing not only the characteristics of the effusion itself but also the surrounding anatomical structures. For instance, pleural thickening or nodules may indicate pleural metastasis, while soft tissue characteristics can signify edematous changes commonly seen in patients with malnutrition or heart failure.

## Discussion

In this study, we utilized TUS images to differentiate between MPE and BPE. We developed a deep-learning model TUSnet to differentiate MPE and BPE based on TUS images from 601 patients and further validated our model in an external testing cohort with 144 patients. The model achieved good performance in the internal testing cohort (AUC 0.814) and the external testing cohort after fine-tuning (AUC 0.753).

Our study is the first to explore the possibility of using TUS images to differentiate MPE and BPE through a deep learning model. There are limited studies in this field and previous studies worked on the identification of pleural effusion and segmentation but not their aetiology [22, 23]. Our model required only a few TUS images for aetiology interpretation, instead video clips, which simplifies future clinical implementation.

We did not classify between transudates and exudates since this is not the focus of our study. Also, Light’s criteria, despite its high accuracy, may still misclassify transudates as exudates and may have limited discrimination ability in pleural effusion with mixed etiologies [24–26]. Moreover, the classification does not bring more evidence than the effusion image or the cytopathology report [1, 2, 18]. Underlying disease was also not implemented in the model to broaden the generalizability in clinical settings.

Image segmentation was not done in our study. Auto-segmentation may aid in the pleural effusion analysis, however it may only focus on the pleural effusion and overlook the surrounding findings such as pleural thickening, nodules or lung consolidation. The attention heatmaps reveal that TUSnet’s predictive rationale relies not only on the pleural effusion itself but also on surrounding anatomical structures. This suggests the model may be detecting morphological changes—such as micro-nodularity, localized pleural thickening or echogenecity—that are clinically associated with malignancy and subtle to human eye.

Our study has several notable strengths. First, there was no protocol to unify the ultrasound machine and settings in our study. Previous medical studies have shown the importance of a standardized setting for deep learning model development. Ultrasound propagation may be affected by factors such as machine, subcutaneous thickness and density, hence a uniformed machine setting adjustment may not be applicable in the real-world setting. We were able to pre-process the images with reshaping, data augmentation, and fine-tuning methods to provide a uniformed image quality and develop this model. The result implies the possibility to provide good generalizability for the real world across different ultrasound machines and settings.

Additionally, no demographic or biomarker information were needed for our model. This advantage makes our model easier to approach as compared with other deep-learning model studies. Hence, our study can be widely applicable to any clinical settings, such as resource-limited sites lacking immediate laboratory facilities for effusion analysis. Moreover, only a few TUS images from each patient were required for analysis, rather than videos, further reducing the requirement for data transmission and remote analysis.

There are limitations to our study. TUS has a lower discrimination ability for malignancy than CXR or chest CT as TUS can only approach the peripheral part of thoracic cavity [13, 27]. TUS is a good positive and bad negative imaging tool, and physicians should not rely solely on it to exclude malignancy. However, we could still generate a good performance in both our internal testing cohort and external testing cohort. Integrating CXR or CT may further increase the model performance and will be our future interest. Second, our model serves as a supportive tool to assist in MPE diagnosis but not a replacement for the cytopathology review of pleural specimens. Cytopathology review remains the gold standard for MPE diagnosis and malignant molecular marker exploration [2, 28, 29]. Our findings highlight the value of this model in optimizing diagnostic workflows. By identifying patients with a high probability of MPE, the tool supports the rationale for further investigation, such as repeat sampling, while reducing the burden of unwarranted procedures in patients with predicted BPE. Moreover, our model may aid in diagnosis in patients who are relatively contraindicated for thoracentesis, such as thrombocytopenia, frailty, and anticoagulation use. Third, the images were selected by pulmonologists who are experienced in TUS. The operator may need a basic knowledge of TUS and malignancy-related findings to select the required image. However, all images in our study were collected following an easy protocol, which only requires a short course of education to develop required skills. Finally, our model lacks a prospective cohort to prove its clinical value. This study represents the first step in establishing the feasibility of a TUS-based deep learning model using a large multicenter retrospective cohort with external validation. Further multicenter prospective study will be required to extend our findings to practical use.

## Conclusions

We developed a deep-learning model for pleural effusion differential diagnosis between MPE and BPE based on TUS images. We further validated our deep-learning model in an independent cohort. To date, this is the first study to successfully implement a deep-learning model using TUS images in differentiating pleural effusion aetiology. Our study provides a non-invasive, convenient, and lab-free method to assist in MPE identification with high accuracy.

## Data Availability

Data are available upon reasonable request from the corresponding author.

## Abbreviations

MPE: Malignant pleural effusion
CXR: Chest radiography
CT: Computed tomography
TUS: Thoracic ultrasound
BPE: Benign pleural effusion
AUC: Area under curve
ROC: Receiver operating characteristic
